# The variant‐specific burden of SARS‐CoV‐2 in Michigan: March 2020 through November 2021

**DOI:** 10.1002/jmv.27982

**Published:** 2022-07-14

**Authors:** Joshua G. Petrie, Marisa C. Eisenberg, Adam S. Lauring, Julie Gilbert, Samantha M. Harrison, Peter M. DeJonge, Emily T. Martin

**Affiliations:** ^1^ Center for Clinical Epidemiology and Population Health Marshfield Clinic Research Institute Marshfield Wisconsin USA; ^2^ Department of Epidemiology University of Michigan School of Public Health Ann Arbor Michigan USA; ^3^ Departments of Internal Medicine and Microbiology and Immunology University of Michigan Ann Arbor Michigan USA; ^4^ Wisconsin Department of Health Services Madison Wisconsin USA

**Keywords:** case fatality, COVID‐19, incidence, infection, SARS‐CoV‐2, seroprevalence

## Abstract

Accurate estimates of the total burden of severe acute respiratory syndrome coronavirus 2 (SARS‐CoV‐2) are needed to inform policy, planning, and response. We sought to quantify SARS‐CoV‐2 cases, hospitalizations, and deaths by age in Michigan. Coronavirus disease 2019 cases reported to the Michigan Disease Surveillance System were multiplied by age and time‐specific adjustment factors to correct for under‐detection. Adjustment factors were estimated in a model fit to incidence data and seroprevalence estimates. Age‐specific incidence of SARS‐CoV‐2 hospitalization, death, vaccination, and variant proportions were estimated from publicly available data. We estimated substantial under‐detection of infection that varied by age and time. Accounting for under‐detection, we estimate the cumulative incidence of infection in Michigan reached 75% by mid‐November 2021, and over 87% of Michigan residents were estimated to have had ≥1 vaccination dose and/or previous infection. Comparing pandemic waves, the relative burden among children increased over time. In general, the proportion of cases who were hospitalized or who died decreased over time. Our results highlight the ongoing risk of periods of high SARS‐CoV‐2 incidence despite widespread prior infection and vaccination. This underscores the need for long‐term planning for surveillance, vaccination, and other mitigation measures amidst continued response to the acute pandemic.

## 1 INTRODUCTION

Coronavirus disease 2019 (COVID‐19) pandemic response has been challenged by rapidly changing circumstances including the emergence of severe acute respiratory syndrome coronavirus 2 (SARS‐CoV‐2) variants and a developing understanding of the breadth and duration of vaccine‐induced immunity. As policy‐makers seek to update decisions in an environment of shifting vaccination and infection patterns, a better understanding of the overall level of population immunity based on best‐available surveillance data is needed. However, accurately estimating the total burden of SARS‐CoV‐2 infection is difficult.

Public health surveillance systems are challenged by persistent under‐detection of cases, particularly for those infections that do not require medical attention. Further, approximately one‐third of all infections may be completely asymptomatic.^1^, ^2^ Under‐detection is also expected to vary by age and over time related to testing availability and testing behaviors.^3^ Seroprevalence studies can be helpful for surveillance and estimating the total burden of infection.^4^, ^5^ However, seroprevalence estimates provide a snapshot of past and recent infections that can be difficult to disentangle and can underestimate the cumulative incidence of infection due to waning antibodies.^6^

The state of Michigan experienced four waves of SARS‐CoV‐2 transmission during the COVID‐19 pandemic through December 2021. Each pandemic wave has affected the general population and healthcare systems in different ways suggesting changing patterns of infection and severity by age. Michigan is also one of few states that experienced substantial transmission of both the SARS‐CoV‐2 Alpha (B.1.1.7 lineage) and Delta (B.1.617.2 lineage) variants.^7^ We sought to quantify the burden of SARS‐CoV‐2 cases, hospitalizations, and deaths by age and geography over time in Michigan by integrating public health surveillance data, serial seroprevalence estimates, and genomic surveillance data. Burden estimates were used to examine how the risk of hospitalization and death varied over time by age and SARS‐CoV‐2 variant.

## 2 METHODS

### 2.1 Cases

Confirmed COVID‐19 cases were those reported to the Michigan Disease Surveillance System (MDSS) with those reported in the Michigan Department of Corrections system excluded. MDSS data were accessed via a data use agreement between the University of Michigan and the Michigan Department of Health and Human Services. The Institutional Review Board at the University of Michigan Medical School reviewed this project and determined it to be exempt from secondary research for which informed consent is not required.

We used a model, adapted from Shioda et al.,^6^ to estimate the cumulative incidence of infection from MDSS case incidence data and Michigan seroprevalence data from the CDC's Nationwide Commercial Lab Seroprevalence study while accounting for waning (Supporting Information Methods: Figure S1).^8^ We estimated case adjustment factors during five time periods (March–May 2020, June–September 2020, October 2020–February 2021, March–May 2021, and June–November 2021) in age group‐specific models (0–17, 18–49, 50–64, and ≥65 years). Parameters were estimated using Markov Chain Monte Carlo sampling; point estimates were taken as the median posterior sample, and 95% credible intervals (CrI) were taken as the 2.5th and 97.5th percentiles. As in Shioda et al., time from illness onset to seroconversion was assumed to follow a Weibull distribution with a mean of 11.5 days and SD of 5.7 days.^9^ We estimated the average time from seroconversion to seroreversion to have a mean of 229.7 days (7.6 months) and SD 105.3 days by fitting a Weibull distribution, using a weighted least squares method, to published data on the duration of seropositivity measured by the Abbott ARCHITECT SARS‐CoV‐2 anti‐nucleocapsid immunoglobulin G immunoassay (Supporting Information: Figure S2).^10^, ^11^ This assay was used in the Nationwide Commercial Lab Seroprevalence study in Michigan.^4^

**Figure 1:**
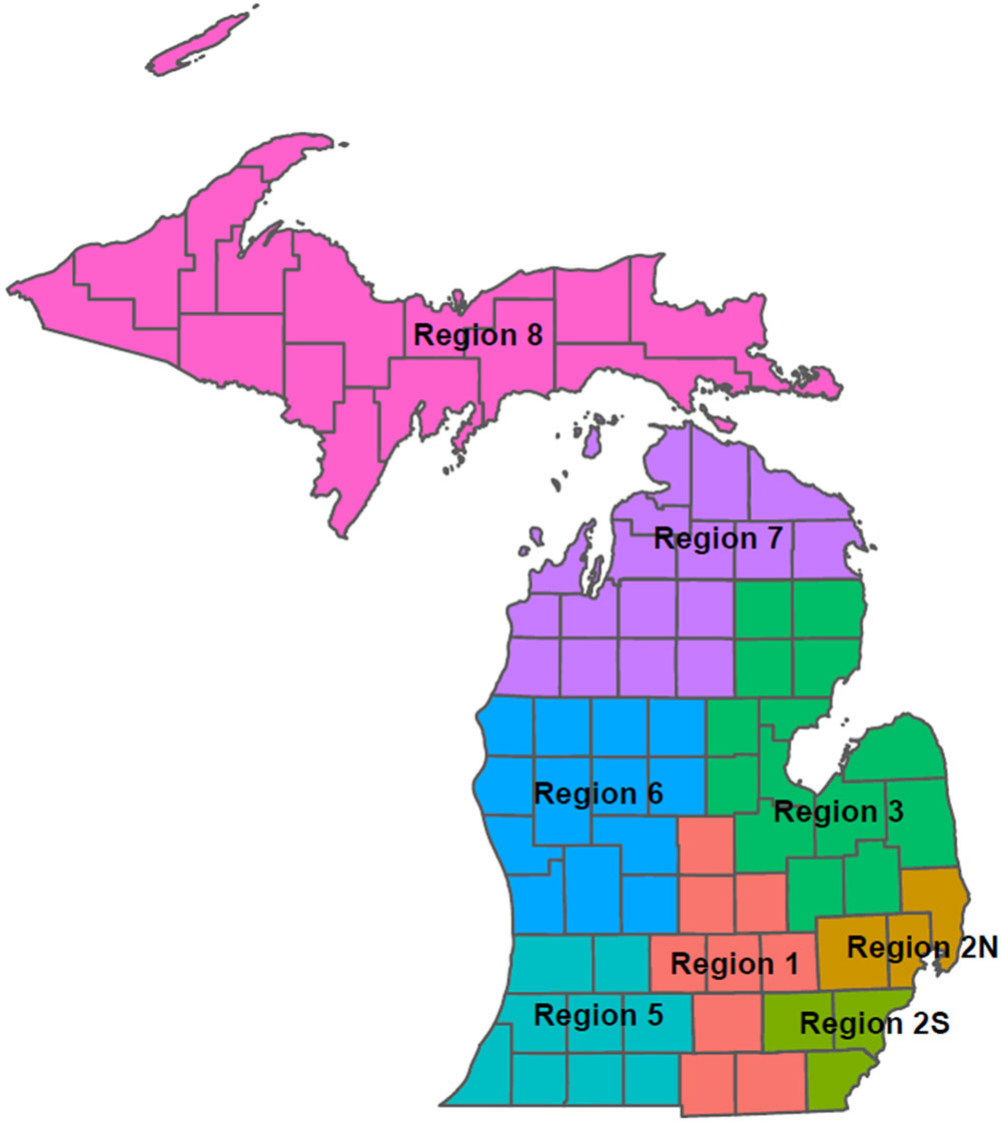
Map of Michigan Public Health Preparedness Regions.

Daily MDSS cases were multiplied by the age‐ and time‐specific case adjustment factors and their 95% CrI to estimate a range of total infections. Adjusted total infections were aggregated by age group and week to state and public health preparedness region (Figure 1). Age groups used throughout this analysis are 0–17, 18–19, 20–29, 30–39, 40–49, 50–59, 60–69, 70–79, and ≥80 years based on age granularity across data sources.

### 2.2 Hospitalizations

Weekly, facility‐level adult and pediatric inpatient admissions for confirmed or suspected COVID‐19 were identified from HHS Protect.^12^ Facilities were mapped to the public health preparedness region, and weekly admission counts were aggregated to state and region levels. Hospitalized cases were also identified from MDSS; because admission status is ascertained at the time of case investigation and individuals may not have been hospitalized yet, these data represent an underestimate of total hospitalizations, but age and geographic data are available. The total number of weekly admissions from HHS Protect was multiplied by week‐ and region‐specific age distributions of hospitalized MDSS cases to estimate total hospitalizations in each age group, region, and week.

### 2.3 Deaths

Weekly estimates of deaths were made using National Center for Health Statistics (NCHS) excess death estimates^13^ and confirmed and probable COVID‐19 deaths reported to MDSS with those reported in the Michigan Department of Corrections system excluded. Weekly Michigan deaths were estimated as the total COVID‐19 deaths reported to MDSS or the upper NCHS estimate of excess deaths, whichever was higher. The total combined MDSS/NCHS weekly deaths were multiplied by the week‐ and region‐specific age distributions of deaths from MDSS to estimate the total deaths in each age group, region, and week.

### 2.4 Variant prevalence

Proportions of Alpha and Delta variant and ancestral lineage SARS‐CoV‐2 viruses among all characterized viruses in Michigan were obtained from covariants.org for 2‐week periods.^14^ Counts of cases, hospitalizations, and deaths were multiplied by the week‐specific variant proportions to estimate cases, hospitalizations, and deaths attributable to Alpha, Delta, and ancestral viruses.

### 2.5 Vaccination

The proportion of the population receiving ≥1 dose of vaccine by age and region was calculated by week from publicly available data reported to the Michigan Care Improvement Registry.^15^ Vaccination data were available by the following age groups: 5–11, 12–15, 16–19, 20–29, 30–39, 40–49, 50–64, 65–74, ≥75 years. Vaccination counts were reassigned to the analysis age groups described under *Cases* as follows. If a vaccination age group spanned two analysis age groups, vaccination counts were assigned to each of the analysis age groups according to the proportion of age years contained in each age group. For example, 2/3 of the vaccinations in the 50–64 year vaccine age group were attributed to the 50–59 year analysis age group and 1/3 to the 60–69 year analysis age group. Vaccination counts from the ≥75 years vaccination age group were assigned to the 70–79 and ≥80 years analysis age groups according to the actual proportion of ≥75‐year‐olds in Michigan who are also ≥80 years old (57%).

### 2.6 Analysis

The 16 highest incidence weeks of Fall 2020, Spring 2021, and Fall 2021 waves of COVID‐19 in Michigan were compared. The Fall 2020 wave was defined from October 11, 2020 through January 30, 2021, the Spring 2021 wave was defined from February 28, 2021 through June 19, 2021, and the Fall 2021 wave was defined from July 25, 2021 through November 13, 2021. Delta transmission in Fall 2021 had not peaked at the time of analysis. Age‐specific estimated cases, hospitalizations, and deaths were plotted and compared across the waves, and by predicted variant status. Age‐ and region‐specific proportions of cases who were hospitalized or died were also compared across waves. The effective reproduction number (*R*_e_) was estimated for Alpha, Delta, and ancestral lineage viruses over rolling 2‐week intervals using the R EpiEstim package with a serial interval of mean 5.68 and SD 4.77 days.^16^, ^17^ All analyses were completed using R (R Foundation for Statistical Computing; version 4.1.1).

## 3 RESULTS

**Figure 2:**
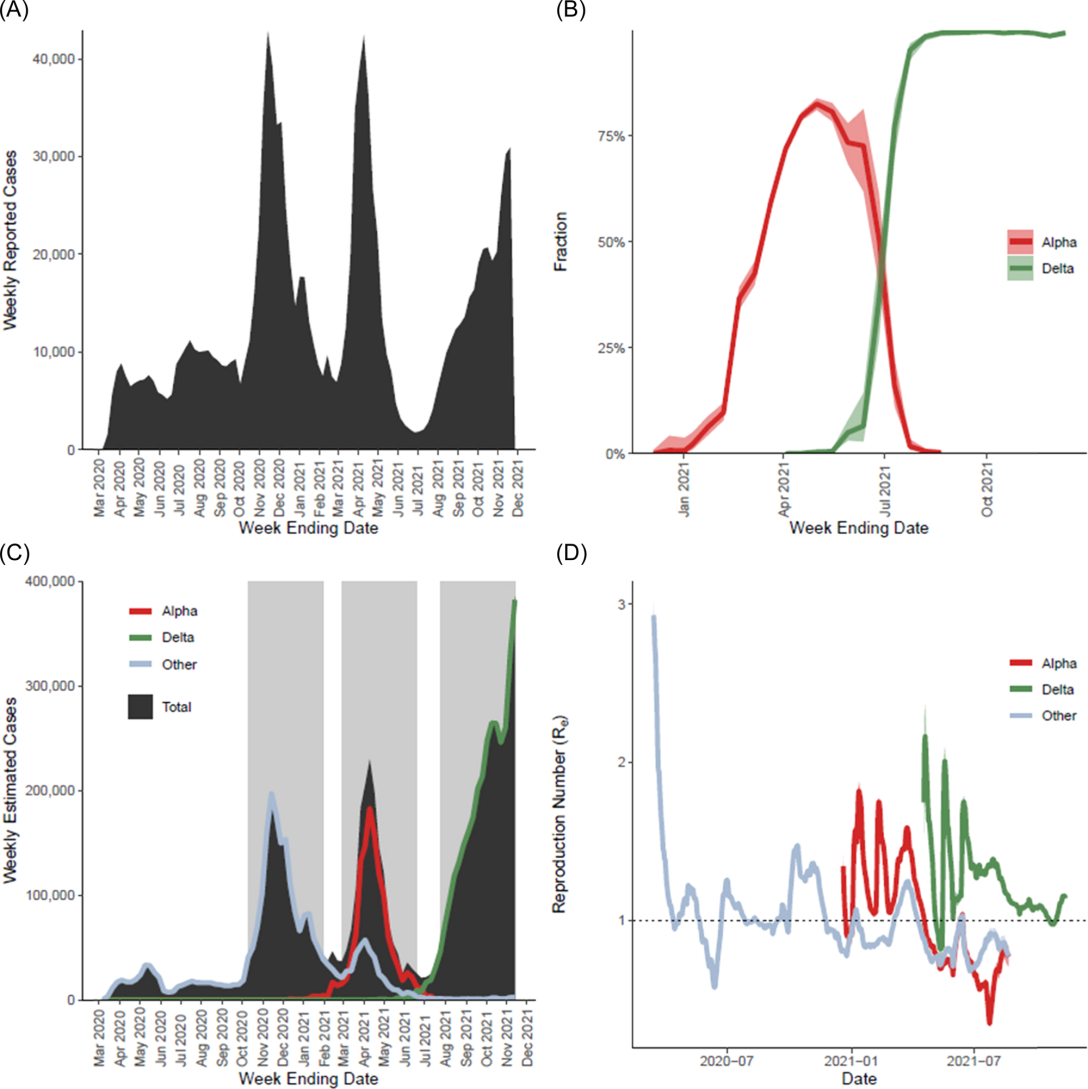
Epidemiology of the SARS‐CoV‐2 pandemic in Michigan. (A) Weekly reported confirmed and probable SARS‐CoV‐2 cases in Michigan. (B) Fraction of Alpha and Delta variant among characterized virus isolates. (C) Epidemic curve of estimated total, Alpha, Delta, and nonvariant SARS‐CoV‐2 cases in Michigan; shaded gray areas highlight periods of analysis comparing Fall 2020 (October 11–January 30), Spring 2021 (February 28–June 19), and Fall 2021 (July 25–November 13) waves. (D) Estimated reproduction number (*R*_e_) comparing Alpha, Delta, and nonvariant viruses. SARS‐CoV‐2, severe acute respiratory syndrome coronavirus 2.

During the Fall 2020 wave, 359 061 confirmed COVID‐19 cases were reported in Michigan; 285 528 confirmed cases were reported in Spring 2021, and 262 258 confirmed cases were reported in Fall 2021 (Figure 2A). After applying age and time‐specific adjustment factors (Supporting Information: Table S1), we estimated that there were 1 649 547 total cases in the Fall 2020 wave, 1 594 954 total cases in Spring 2021, and 3 329 748 total cases in Fall 2021. Alpha variant viruses were first detected in December 2020 (Figure 2B). The proportion of sequenced viruses identified as Alpha rapidly increased to nearly 50% by late February, and further increased to approximately 75% by early April. Delta variant viruses emerged in Michigan in April 2021 and accounted for nearly 100% of cases by mid‐July 2021. Applying the estimated variant proportions to the estimated total weekly case time series, we estimated that there were 1 178 658 (74%) Alpha variant cases and 397 379 (25%) ancestral lineage cases in the Spring 2021 wave (Figure 2C). Ancestral lineage and Delta variant cases accounted for over 99% of infections in the Fall 2020 and Fall 2021 waves, respectively. The reproduction number for Alpha variant viruses was 12% (95% CrI: 12%, 13%) higher than that of ancestral viruses, and the reproduction number for Delta variant viruses was 91% (95% CrI: 91%, 92%) higher than that of Alpha variant viruses averaged over periods with overlapping circulation (Figure 2D).

**Figure 3:**
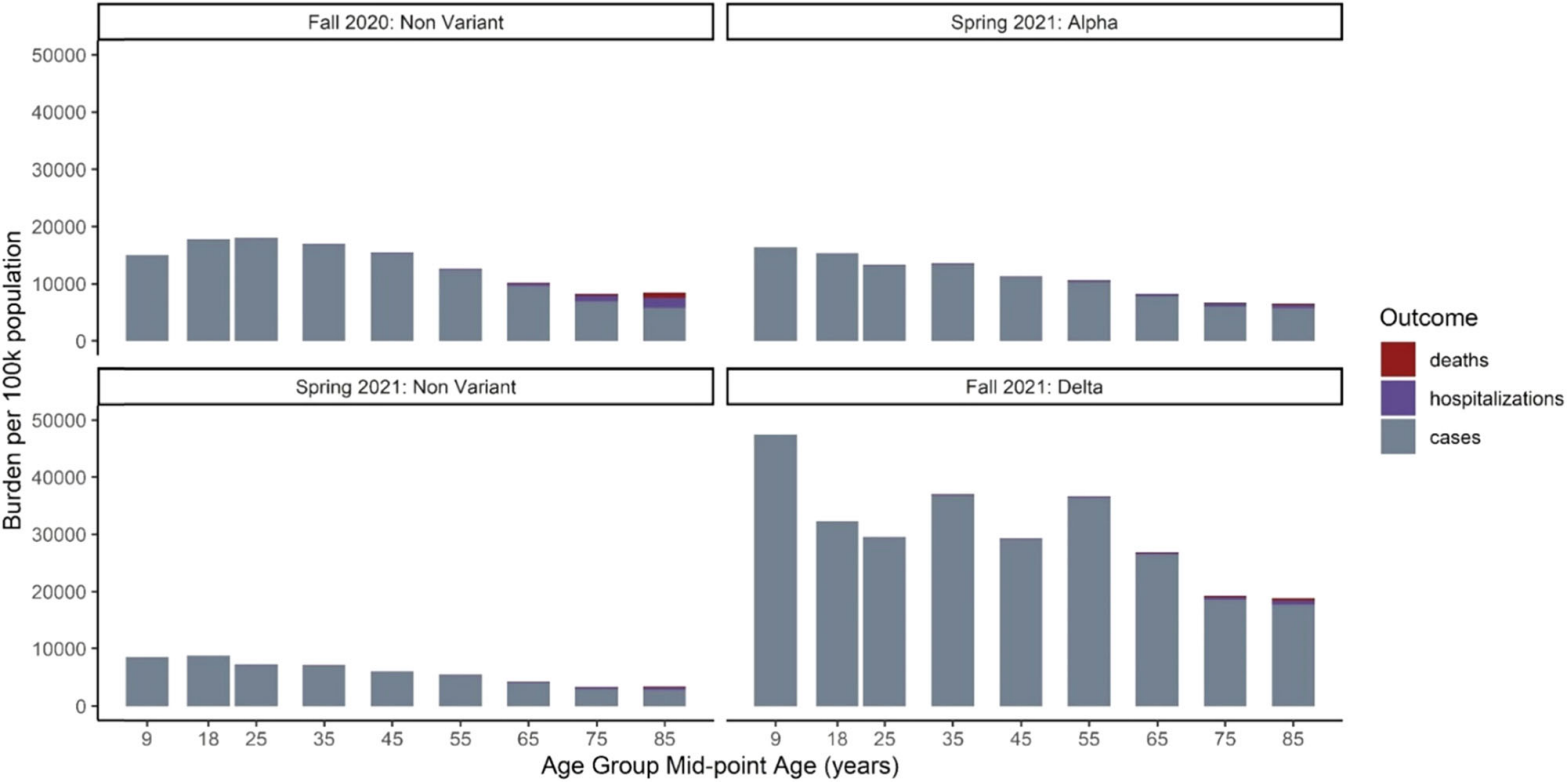
Estimated SARS‐CoV‐2 burden per 100 000 population by age in Michigan comparing estimated nonvariant infections in Fall 2020 (October 11–January 30), Alpha variant and nonvariant infections in Spring 2021 (February 28–June 19), and Delta variant infections in Fall 2021 (July 25–November 13). The total height of the bars is the total number of cases in each age group that is, hospitalizations and deaths were subtracted from the case counts. However, we do not know the proportion of hospitalized cases that died or the proportion of deaths that were not hospitalized so the combined height of those segments overestimates the total number of severe outcomes. SARS‐CoV‐2, severe acute respiratory syndrome coronavirus 2.

Compared with the Fall 2020 wave in Michigan, there was a higher burden of infection among the youngest age group, but a lower burden among the over 60 year age groups in Spring 2021 (Figure 3). The relationship between burden and age appeared similar comparing Alpha and nonalpha variant infections during Spring 2021; Alpha variant infections consistently accounted for approximately 2/3 of the total infections in each age group. The Fall 2021 Delta variant wave had substantially higher numbers of infections than the two prior waves. Incidence was high in all age groups, but young children and middle‐aged adults were most heavily affected. In all waves, hospitalization and death were unlikely in the youngest age groups; individuals over 60 were most likely to be hospitalized and die in all waves.

**Figure 4:**
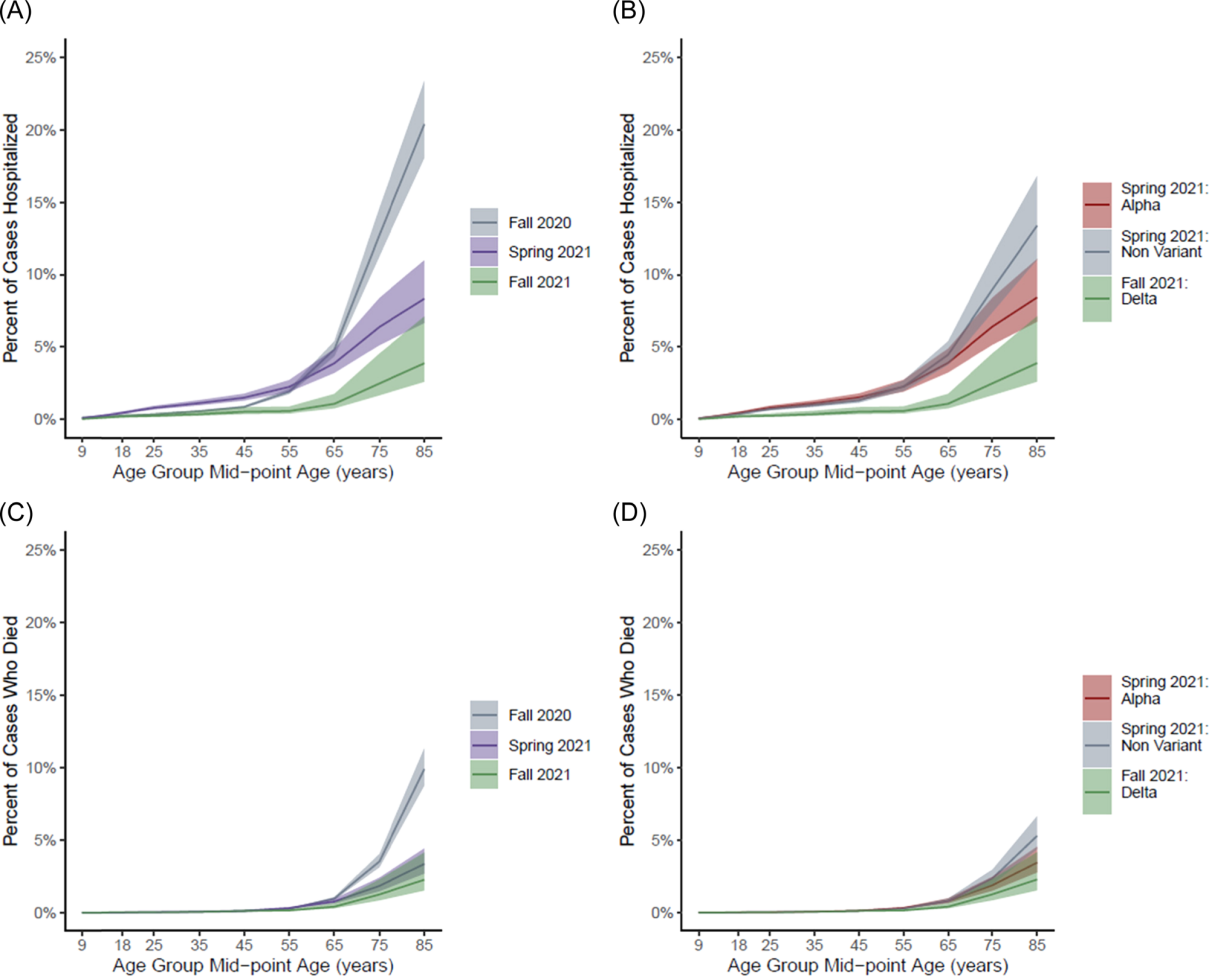
Percent of SARS‐CoV‐2 cases in Michigan who were hospitalized (A) comparing Fall 2020 (October 11–January 30), Spring 2021 (February 28–June 19), and Fall 2021 (July 25–November 13) waves, and (B) comparing estimated Alpha variant and non‐variant infections in Spring 2021, and Delta variant infections in Fall 2021. Percent of SARS‐CoV‐2 cases in Michigan who died (C) comparing Fall 2020, Spring 2021, and Fall 2021 waves, and (D) comparing estimated Alpha variant and nonvariant infections in Spring 2021, and Delta variant infections in Fall 2021. SARS‐CoV‐2, severe acute respiratory syndrome coronavirus 2.

In all waves, the proportion of cases who were hospitalized and the proportion of cases who died in Michigan increased substantially with age. Those in the middle age groups (20–29 through 40–49 years) were more likely to be hospitalized if infected in Spring 2021 than if infected in Fall 2020 (Figure 4A). However, this did not appear to be Alpha variant‐specific; the likelihood of hospitalization did not vary by lineage in any age group in the spring (Figure 4B). Cases who were ≥80 years were more likely to be hospitalized (20% [95% CrI: 18%, 23%] vs. 8% [95% CrI: 7%, 11%] vs. 4% [95% CrI: 3%, 7%]) and to die (10% [95% CrI: 9%, 11%] vs. 3% [95% CrI: 3%, 4%] vs. 2% [95% CrI: 2%, 4%]) if infected in Fall 2020 than if infected in Spring 2021 or Fall 2021 (Figure 4A,C). This also did not appear to be driven by Alpha variant circulation in Spring 2021 as the proportion of cases that died was similar comparing Alpha and ancestral infections (Figure 3D). Across all age groups, those with Delta variant infection in Fall 2021 were less likely to be hospitalized or die than those with ancestral or Alpha variant infections in previous waves.

**Figure 5:**
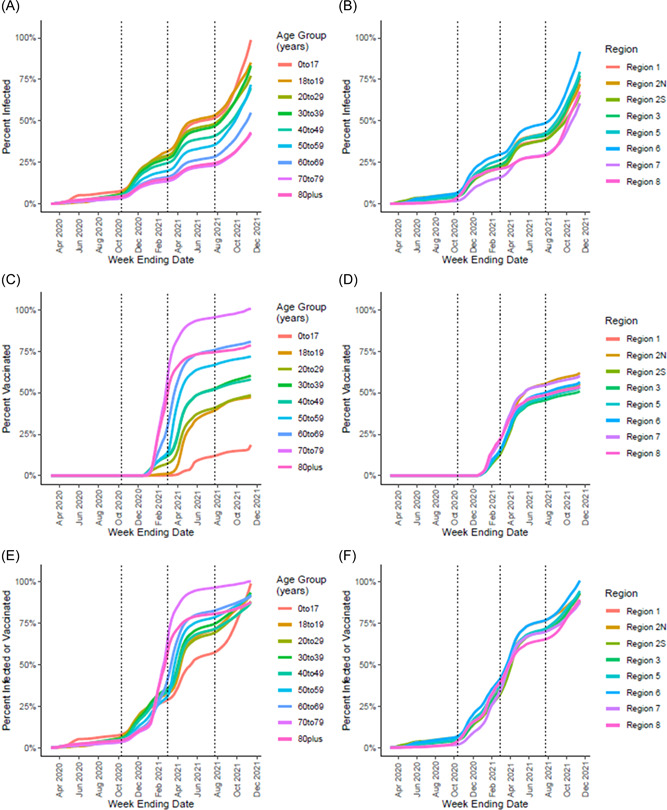
Cumulative percentage of Michigan population with SARS‐CoV‐2 infection, vaccination, or both by age group (A, C, E) and geographic region (B, D, F). Dotted vertical lines represent the start of the Fall 2020 (October 11–January 30), Spring 2021 (February 28–June 19), and Fall 2021 (July 25–November 13) waves. SARS‐CoV‐2, severe acute respiratory syndrome coronavirus 2.

Cumulative incidence by age group was tightly clustered between 3% and 8% just before the Fall 2020 wave, with the 0–17 year age group most likely (8%) and the 70–79 year age group least likely to have been previously infected (3%) (Figure 5A). Following the Fall 2020 wave, there was a wider range of age‐specific cumulative incidence (13%–28%) that generally decreased with increasing age. The 18–19 year age group was now most likely (28%), the 70–79 year age group remained least likely to have been infected (13%), and approximately 22% of the total population had been infected before the Spring 2021 wave. Following the Spring 2021 wave, the overall cumulative incidence was 40% and ranged from 22% to 52% by age group. By November 12, 2021, the overall cumulative incidence was 75% and ranged from 99% to 42% by age group. Cumulative incidence of infection did not vary as much by Michigan Public Health Preparedness Region as it did with age. However, the more rural Regions 7 and 8 consistently had the lowest cumulative incidence throughout the pandemic (Figure 5B).

Substantial vaccine uptake occurred among the 60–69, 70–79, and 80+ year age groups in early 2021, with an estimated 47%, 55%, and 28% having ≥1 dose of vaccine, respectively, before the Spring 2021 wave (Figure 5C). Vaccination coverage increased in all age groups throughout Spring 2021 before slowing in the summer months, with final coverage ranging from >99% among 70–79‐year‐olds to 19% among 0–17‐year‐olds. Vaccination coverage did not vary substantially by Public Health Preparedness Region, but uptake was initially faster in Regions 7 and 8 possibly reflecting an older population on average (Figure 4D). Proportions receiving ≥1 vaccine dose or previous infection by the end of Spring 2021 ranged from 96% among 70–79‐year‐olds to 55% among 0–17‐year‐olds and followed the same patterns as vaccination coverage (Figure 4E,F). By November 13, 2021, our model estimates that over 87% of Michigan residents in all groups had at ≥1 vaccine dose and/or previous infection.

## 4 DISCUSSION

We estimated substantial under‐detection of SARS‐CoV‐2 infection in Michigan that varied by age and time. Under‐detection among children was highest before May 2020 with an estimated 83 infections for every reported case. Detection improved to six infections per report in the summer of 2020 before gradually worsening to 25 infections per report after June 2021. In contrast, infections among adults were much more likely to be detected throughout the pandemic, but under‐detection increased over time with the highest numbers of infections per report (8–14) after June 2021. Accounting for under‐detection, we estimate the cumulative incidence of infection in Michigan reached 75% by mid‐November 2021. Further accounting for vaccination, we estimate the vast majority of Michiganders across all age groups had antigenic exposure to SARS‐CoV‐2 by mid‐November 2021. These estimates can inform response and planning, for example, anticipating the scale of support services needed for individuals with postacute COVID‐19 symptoms.

Following initial Alpha variant introductions, Michigan experienced a surge of infections in Spring 2021 when many US states had declining incidence.^7^ We estimate that Alpha variant infections accounted for approximately two‐thirds of all infections during Michigan's Spring 2021 wave. Consistent with the observed rapid replacement, we estimated the Alpha *R*_e_ was 12% higher than that of ancestral viruses. Alpha variant viruses were rapidly replaced by Delta in summer 2021, and we estimated that the Delta *R*_e_ was almost twice that of Alpha in periods of cocirculation. These estimates are somewhat lower than previous reports that found Alpha to be 40%–100% more transmissible than ancestral SARS‐CoV‐2 viruses,^18^, ^19^ but similar to reports of increased transmissibility of Delta relative to Alpha.^20^, ^21^

In the time since this analysis was carried out, Delta infections continued to rise in Michigan before being rapidly overtaken by Omicron resulting in record high daily infections. Michigan's experience in the winter of 2022 makes it clear that combined levels of prior infection and vaccination that exceed 80% are not sufficient to reach herd immunity. Suboptimal vaccine coverage, waning of natural and vaccine‐induced immunity, and the emergence of more transmissible variants have facilitated ongoing transmission.^22^, ^23^, ^24^ Indeed, it is unlikely that true herd immunity will be reached entirely ending SARS‐CoV‐2 transmission just as descendants of the 1968 and 2009 influenza pandemics continue to circulate today. This underscores the need for long‐term planning in policy, public health capacity, and research priorities. These results also suggest that nonpharmaceutical mitigation measures may be needed during times of high transmission going forward.

We observed that the proportion of cases who were hospitalized or died decreased with each subsequent wave. Because of the ecologic nature of this analysis, we are unable to attribute reduced severity to a specific cause. However, our results do not suggest differences in the severity of Alpha and ancestral viruses in Spring 2021. Although differential severity has not been conclusively demonstrated, some studies have estimated Alpha and Delta are more severe than ancestral SARS‐CoV‐2, at least among unvaccinated individuals.^25^, ^26^ COVID‐19 vaccines have been effective in preventing severe outcomes of infection.^27^, ^28^, ^29^ This suggests that at least through the emergence of Delta, age‐specific reductions in severity were likely due to vaccination and improved treatment options over time. Despite apparent reductions in severity, the emergence of more transmissible variants has stressed healthcare systems through large patient volumes and infections among healthcare workers.

A notable exception to the general trend of decreasing severity is that adults <50 years were at increased risk of hospitalization in Spring 2021 relative to Fall 2020. It is possible that patterns in vaccine uptake could confound differences in severity over time. Among those prioritized for early vaccination, younger adults with chronic conditions had a similar low intention to vaccinate as “essential workers” generally.^30^ Racial disparities in both vaccine uptake and severe outcomes of SARS‐CoV‐2 have also been demonstrated.^31^, ^32^ If healthier adults were more likely to be vaccinated earlier, the remaining susceptible population may have been more likely to be hospitalized given infection. Factors associated with vaccine uptake warrant further investigation and consideration in analyses of differential severity.

Our results should be interpreted in the context of multiple limitations. (1) We were unable to account for reinfection, and vaccination was assumed to be independent of prior infection. (2) We relied on Michigan‐specific data from the Nationwide Commercial Lab Seroprevalence study to calculate case adjustment factors to correct for under‐detection. That study has its own limitations,^4^, ^8^ and the representativeness of its sample to the Michigan population as a whole is unclear. (3) Case adjustment factors were sensitive to antibody waning rate assumptions. We found that the average time from seroconversion to seroreversion could not be estimated simultaneously with the adjustment factors due to identifiability issues, so we specified the former parameter from existing literature. (4) The case adjustment factors provided our main source of uncertainty. There are other sources of uncertainty that we were unable to propagate or account for. (5) SARS‐CoV‐2 sequence data reported to GISAID may not reflect true community variant proportions, particularly shortly after emergence when sampling may be biased toward outbreaks.

## 5 CONCLUSIONS

Our results highlight the ongoing risk of SARS‐CoV‐2 infection despite widespread prior infection and vaccination in the population. This underscores the need for long‐term planning for surveillance, vaccination, and other mitigation measures amidst continued response to the acute pandemic. The multiple streams of data on case incidence, infection outcomes, vaccine uptake, and genomic characterization that have facilitated the ongoing COVID‐19 pandemic response should be leveraged to inform response and updates to the SARS‐CoV‐2 vaccine composition and delivery schedule.

## AUTHOR CONTRIBUTIONS

Joshua G. Petrie conceived and designed the analysis, performed the analysis, led manuscript writing, and supervised the project. Marisa C. Eisenberg contributed to the analysis and interpretation of results, facilitated the acquisition of data, and edited the manuscript. Adam S. Lauring contributed data on genetic characterization of severe acute respiratory syndrome coronavirus 2 variants, and edited the manuscript. Julie Gilbert, Samantha M. Harrison, and Peter M. DeJonge wrote code used in the analysis, assisted with data acquisition, and edited the manuscript. Emily T. Martin conceived and designed the analysis, edited the manuscript, and supervised the project.

## CONFLICT OF INTEREST

The authors declare no conflict of interest.

## Supporting information

Supplementary information.

## Data Availability

Aggregated data and code that support the findings of this study are openly available at https://github.com/jgpetrie/MI_COVID_burden.
