# Supplementary material for: The variant‐specific burden of SARS‐CoV‐2 in Michigan: March 2020 through November 2021
Source: J Med Virol. 2022 Jul 14;94(11):5251–9. doi: 10.1002/jmv.27982 (PMC9350192; doi:10.1002/jmv.27982)
Supplement: Supplementary file 1 — Supplementary information. [file JMV-94-5251-s001.docx]

**SUPPLEMENT**

**Supplemental Methods**

We used a model, adapted from Shioda et al., to estimate the true cumulative incidence of infection using COVID-19 case data reported to the Michigan Disease Surveillance System (MDSS) and Michigan seroprevalence data from the CDC's Nationwide Commercial Lab Seroprevalence study while accounting for waning antibody^1,2^. Shioda et al. assumed a constant infection fatality ratio over their period of observation, and used the model to estimate this parameter in order to calculate the true number of infections given observed numbers of cases and deaths. We modified the model to estimate 5 period-specific scaling parameters (ρ_1_ – ρ_5_) that could be used to directly multiply observed case reports and allow infection fatality ratios to vary over time. Scaling parameters were estimated for the following periods: from March 1 to June 1, 2020 (ρ_1_); from June 1 to September 30, 2020(ρ_2_); from October 1, 2020 to February 28, 2021(ρ_3_); from March 1 to May 31, 2021 (ρ_4_); and from June 1 to November 13, 2021 (ρ_5_).

Following Shioda et al., we estimated the number of seropositive individuals (S_t_) on each day of observation, $t$, as the true number of infections with illness onset on each previous day, $k$, multiplied by the probability that they are seropositive on day $t$:

$S_{t}=\sum_{k=1}^{t-1} I_{k}\sum_{y=1}^{t-k} \left\{ g\left( y \right)*\left[ 1-Z\left( t-\left( k+y \right) \right) \right] \right\},2\leq t\leq tmax,1\leq k\leq t-1$.

where $I_{k}$ is the true number of SARS-CoV-2 infections on day $k$ calculated as the observed number of confirmed infections reported to MDSS with illness onset on day $k$ multiplied by the period specific scaling parameter. The probability that an individual infected on day $k$ is seropositive on day $t$ is specified by the second half of equation above: $\sum_{y=1}^{t-k} \left\{ g\left( y \right)*\left[ 1-Z\left( t-\left( k+y \right) \right) \right] \right\}$. Where $g$ is the probability density function of the Weibull distribution of time from symptom onset to seroconversion. As in Shioda et al. we assumed this distribution had a mean of 11.5 days and standard deviation of 5.7 days^3^. $Z$ is the cumulative density function of the Weibull distribution of time from seroconversion to seroreversion. Shioda et al. estimated the mean time from seroconversion to seroreversion from their data, but we found we could not simultaneously estimate this parameter along with the period-specific scaling parameters due to identifiability issues. Instead, we separately estimated the average time from seroconversion to seroreversion to have a mean 229.7 days (7.6 months) and SD 105.3 days by fitting a Weibull distribution, using a weighted least squares method, to published data on the duration of seropositivity measured by the Abbott ARCHITECT SARS-CoV-2 anti-nucleocapsid IgG immunoassay (Supplemental Figure 2)^4,5^. This assay was used in the Nationwide Commercial Lab Seroprevalence study in Michigan^2^. The estimated numbers of true infections and seropositive individuals were constrained such that they could not exceed the total population size. Seroprevalence was calculated as the estimated number of seropositive individuals divided by the estimated cumulative incidence of infection on day $t$.

The 5 period-specific scaling parameters were estimated by comparing the simulated proportion seropositive to that observed in Michigan by the CDC's Nationwide Commercial Lab Seroprevalence study. CDC's Nationwide Commercial Lab Seroprevalence study seroprevalence estimates were produced approximately every 2 weeks from July 30, 2020 through July 9, 2021, and monthly from September through November 2021; no estimates were made between July 10 and September 5, 2021. For each round of the CDC's Nationwide Commercial Lab Seroprevalence study, the log-likelihood was calculated comparing the estimated and observed seroprevalence. The overall log-likelihood was calculated as the sum of the log-likelihoods calculated for each Nationwide Commercial Lab Seroprevalence study round. The log-likelihood was calculated according to the binomial distribution:

$$log-likelihood= \sum_{t=1}^{k} \binom{n_{k}}{x_{k}}{p_{k}}^{x_{k}}{(1-p_{k})}^{(n_{k}-x_{k})}$$

Where $n$ is the number sampled in each CDC seroprevalence sample, $x$ is the number seropositive in each CDC sample, and $p$ is the model estimated seroprevalence as of the date, $t$, of the CDC seroprevalence sample.

The calculated overall log-likelihood was used in Markov chain Monte Carlo (MCMC) analysis to estimate the period-specific scaling parameters. A random-walk Metropolis-Hastings algorithm was used to sample new candidate parameter values from a normal distribution centered at the value of the current parameter value. Separate models were run to estimate period-specific scaling parameters for the following age groups: 0 to 17, 18 to 49, 50 to 64, and ≥65 years. For each age-group-specific model, we ran the MCMC algorithm for 50,000 iterations with a 10,000 iteration burn in. Convergence was assessed based on visual assessment of trace plots of the posterior samples. Parameter point estimates were taken as the median of the posterior sample, and 95% credible intervals (CrI) were taken as the 2.5th and 97.5th percentiles of the posterior sample.

Code and aggregate data used in this study are available at: <https://github.com/jgpetrie/MI_COVID_burden>.

**Supplemental Figure 1. Estimated seropositivity over time among A) 0 to 17; B) 18 to 49; C) 50 to 64; and D) ≥65 year olds in Michigan (black line, 95% credible interval: gray area). Estimates and 95% confidence intervals from the CDC Nationwide Commercial Laboratory Seroprevalence Survey are in red.**


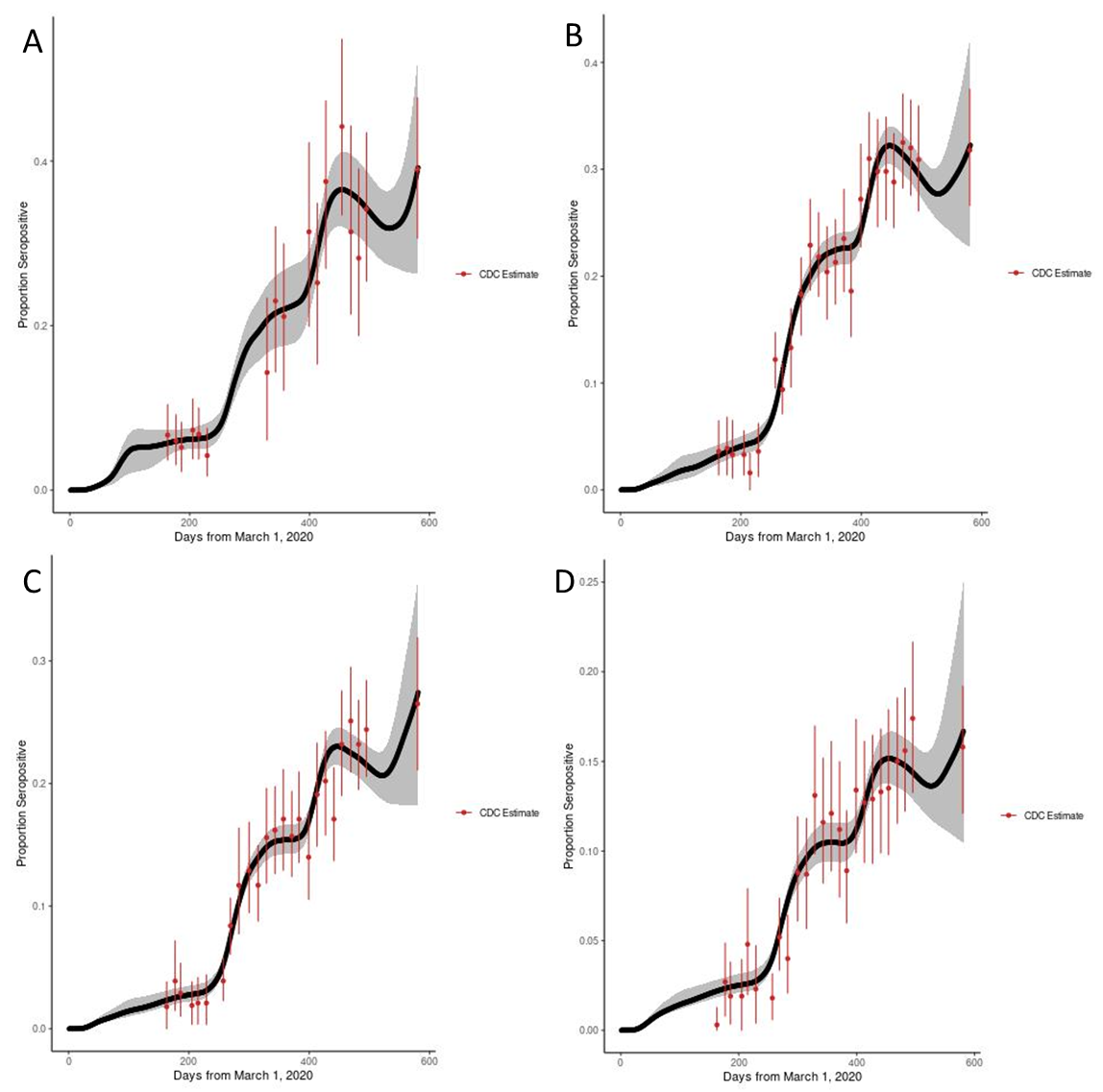


**Supplemental Figure 2. Probabability of detectable antibody to nucleocapsid protein by time following SARS-CoV-2 invection.**


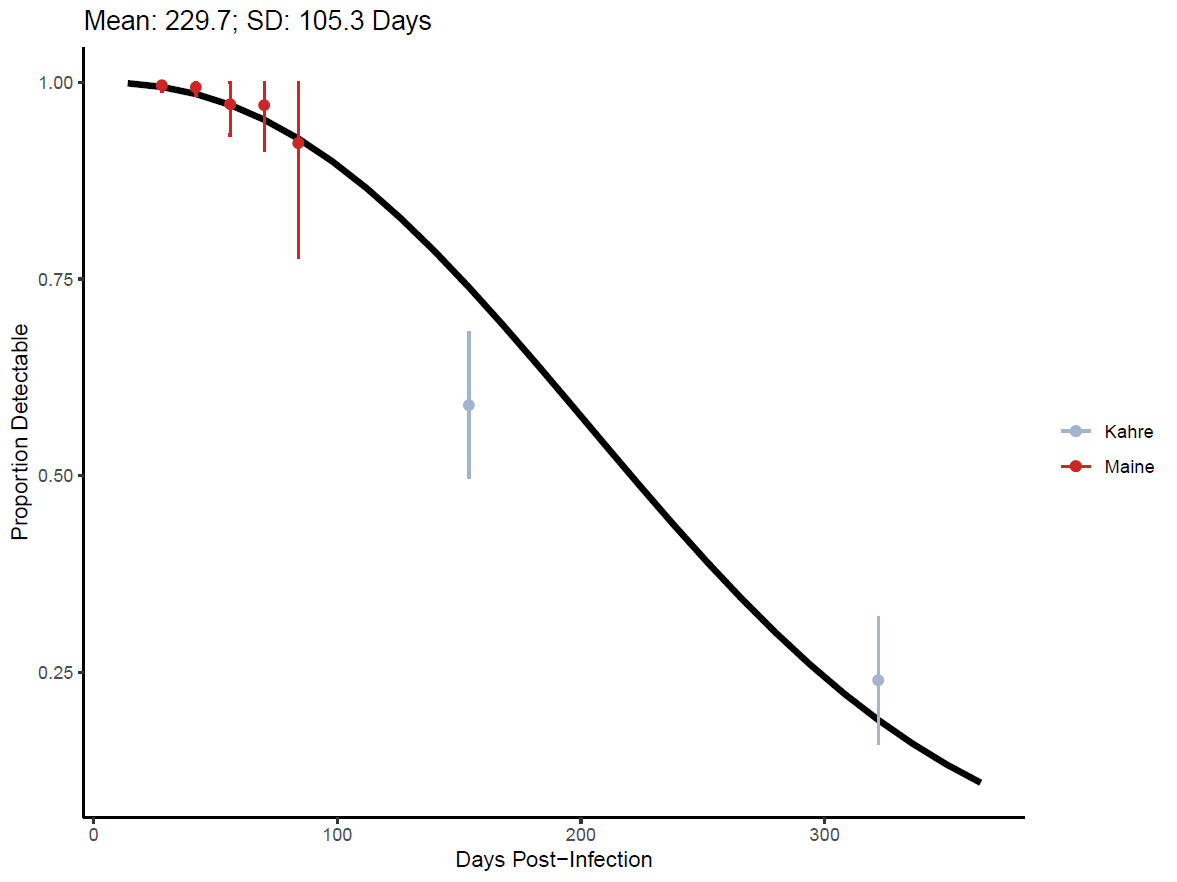


**Supplemental Table 1. Case adjustment factors estimated from incidence and seroprevalence data.**

|  | MAR 2020 –  MAY 2020 | JUN 2020 –  SEP 2020 | OCT 2020 –  FEB 2021 | MAR 2021 –  MAY 2021 | JUN 2021 –  NOV 2021 |
| --- | --- | --- | --- | --- | --- |
| 0 to 17 years | 83.0 (35.3, 119.7) | 5.7 (1.3, 15.1) | 15.0 (11.3, 18.9) | 11.5 (7.8, 15.1) | 25.1 (12.6, 38.2) |
| 18 to 49 years | 2.1 (1.1, 3.7) | 1.4 (1.0, 2.1) | 4.3 (3.9, 4.6) | 4.3 (3.7, 4.9) | 9.8 (6.1, 13.7) |
| 50 to 64 years | 1.5 (1.0, 2.4) | 1.2 (1.0, 1.8) | 3.5 (3.2, 3.8) | 4.6 (3.8, 5.3) | 14.4 (9.3, 19.9) |
| 65+ years | 1.2 (1.0, 1.9) | 1.2 (1.0, 1.8) | 2.5 (2.2, 2.9) | 4.9 (3.8, 6.0) | 7.6 (4.2, 11.4) |

**Supplemental References**

1. Shioda K, Lau MSY, Kraay ANM, et al. Estimating the Cumulative Incidence of SARS-CoV-2 Infection and the Infection Fatality Ratio in Light of Waning Antibodies. *Epidemiology*. 2021;32(4):518-524. doi:10.1097/EDE.0000000000001361

2. Centers for Disease Control and Prevention. Nationwide COVID-19 Infection-Induced Antibody Seroprevalence (Commercial laboratories). Centers for Disease Control and Prevention. Published March 28, 2020. Accessed February 14, 2022. https://covid.cdc.gov/covid-data-tracker/#national-lab

3. Iyer AS, Jones FK, Nodoushani A, et al. Dynamics and significance of the antibody response to SARS-CoV-2 infection. *medRxiv*. Published online July 20, 2020:2020.07.18.20155374. doi:10.1101/2020.07.18.20155374

4. Maine GN, Lao KM, Krishnan SM, et al. Longitudinal characterization of the IgM and IgG humoral response in symptomatic COVID-19 patients using the Abbott Architect. *J Clin Virol*. 2020;133:104663. doi:10.1016/j.jcv.2020.104663

5. Kahre E, Galow L, Unrath M, et al. Kinetics and seroprevalence of SARS-CoV-2 antibodies: a comparison of 3 different assays. *Sci Rep*. 2021;11(1):14893. doi:10.1038/s41598-021-94453-5
